# The current state of registries for participant recruitment in dementia research and the future direction to fast‐track dementia research globally: perspectives from the Alzheimer's Prevention Registry programs

**DOI:** 10.1002/alz70860_098469

**Published:** 2025-12-23

**Authors:** Nellie M. High, Cassandra L Ochoa, Hayley Salata, David Gordon, Jessica B. Langbaum

**Affiliations:** ^1^ Banner Alzheimer's Institute, Phoenix, AZ, USA

## Abstract

**Background:**

The Alzheimer's Prevention Registry (APR) and its GeneMatch program, are web‐based participant recruitment registries to support awareness, interest, and participation in Alzheimer's‐focused studies. The programs complement and supplement researchers’ local recruiting efforts by connecting them with potential study participants. To accomplish this, concerted efforts are required to continually enroll volunteers reflective of the general population, maintain their engagement in the program and interest in research participation, and offer a range of study opportunities for the members.

**Methods:**

The APR is open to adults age 18+; GeneMatch is open to US‐based adults ages 50‐90 who self‐report not having a diagnosis of cognitive impairment. At enrollment in either program, individuals provide their name, contact information, and limited demographic information. After consenting to GeneMatch, participants are sent a buccal swab kit for them to self‐swab and return the completed swab to the CAP/CLIA‐accredited lab for DNA extraction and *APOE* genotyping. GeneMatch does not return the *APOE* results to participants. Concerted efforts have focused on research to understand motivators and facilitators of joining a registry and developing evidence‐based strategies to communicate the importance of joining a registry to currently under‐enrolled and understudied populations, mainly Black and Hispanic adults and males.

**Results:**

The APR launched in May 2012; as of January 2025, >385,950 have joined, mean age 68, 76% women. To date, the APR has helped 165 studies with their recruitment efforts. GeneMatch began recruitment in November 2015; as of January 2025, 117,524 have consented. To date, GeneMatch has helped 29 studies with recruitment. Our related research has found that the general public is not familiar with the term “registry,” and people *already enrolled in registries* do not know how to describe either their involvement or the purpose of the registry; social media algorithms may be driving the disparity in enrollment between men and women; and features of effective messages include diverse racial/ethnic representation in imagery and attitudinal content focused on helping others and advancing science.

**Conclusions:**

Registries can be effective solutions to efficiently connect volunteers to studies. Key successes, challenges, and opportunities for global collaboration will be discussed.

